# Titanium-Enriched Slag Prepared by Atmospheric Hydrochloric Acid Leaching of Mechanically Activated Vanadium Titanomagnetite Concentrates

**DOI:** 10.3390/ma14226736

**Published:** 2021-11-09

**Authors:** En-Hui Wu, Yin-He Lin, Jun Liu, Zhe Wang, Jin-Chuan Liu, Guo-Liang Yin, Jing-Wei Li, Xiang-Kui Cheng, Yu-Long Jia

**Affiliations:** 1 Panzhihua International Research Institute of Vanadium and Titanium, Panzhihua University, Panzhihua 617000, China; wuenhui1026@126.com (E.-H.W.); chengxiangkui2021@163.com (X.-K.C.); 2 School of Materials and Chemical Engineering, Yinbin University, Yibin 644007, China; liujinchuan123@yeah.net (J.-C.L.); y19881131984@163.com (G.-L.Y.); yljia2017@126.com (Y.-L.J.); 3 Titanium Chloride Slag Plant, Sichuan Lomon Mining and Metallurgy Co. Ltd., Panzhihua 617000, China; lyqlyqliujun@163.com; 4 State Key Laboratory of Advanced Metallurgy, University of Science and Technology Beijing, Beijing 100083, China; zhewang@ustb.edu.cn; 5 School of Materials Science and Engineering, Hefei University of Technology, Hefei 230009, China; 6 National-Local Joint Engineering Research Centre of Nonferrous Metals and Processing Technology, Hefei University of Technology, Hefei 230009, China

**Keywords:** vanadium titanomagnetite concentrates, mechanical activation, hydrochloric acid Leaching, titanium-enriched slag, TiO_2_ extraction

## Abstract

The titanium-enriched slag was obtained via atmospheric hydrochloric acid leaching of mechanically activated vanadium titanomagnetite concentrates (VTMCs). Under the influence of mechanical activation, specific physicochemical changes were observed via X-ray diffractometry, scanning electron microscopy, and granulometric laser diffraction analysis. Experimental findings revealed that the mechanical activation of VTMCs resulted in a decrease in the median volume particle diameter (d50) and an increase in the specific surface area (SA) with an increased milling time. The results of the leaching experiment revealed that the mechanical activation treatment favors the extraction of iron (Fe) and titanium dioxide (TiO_2_) from the VTMCs. The Fe and TiO_2_ extractions from the mechanically activated sample after 10 h compared with the unactivated sample were increased by 12.82% and 4.73%, respectively. The presence of the ilmenite phase in the titanium-enriched slag was confirmed by X-ray diffractometry and EDS patterns, and the content of the TiO_2_ in the enriched slag can get as high as 43.75%.

## 1. Introduction

The vanadium-bearing titanomagnetite (VBTM) ore deposit in the Panzhihua–Xichang region, China, is a complex ore that contains many valuable elements, including titanium (Ti), vanadium (V), iron (Fe), chromium (Cr), scandium (Sc), and gallium (Ga), and has high utilization value [1,2,3]. Vanadium titanomagnetite concentrates (VTMCs) and ilmenite concentrates are obtained via an enrichment process from the VBTM ore. The ilmenite concentrates are used as raw materials in producing titanium dioxide (TiO_2_) pigment [4,5]. For many years, the blast furnace process has been employed as an advanced smelting strategy for processing VTMCs [6,7,8].

However, in the blast furnace process, both the Fe and V in VTMCs are reduced to hot metal, while most of the Ti remains unreduced, subsequently forming the Ti-bearing blast furnace slag [9,10]. The Ti slag comprises varying content of TiO_2_ in the range of 22 wt.% to 25 wt.%, and there have been no known effective means of treating the slag [11,12]. Thus, this Ti slag, which is a valuable resource, becomes waste polluting the environment. Consequently, the direct reduction–electric furnace smelting process was developed, and the industrialization test was conducted [13]. The advantage of the direct reduction–electric furnace smelting process is that the Ti resources from the VTMCs can be recycled, but due to the high processing costs compared to the blast furnace process, its industrialization has not been extensively explored [14].

Existing research on the utilization of VTMCs is still focused on the direct reduction–electric furnace smelting process in which the technological parameters of the direct reduction process are optimized by pre-oxidation and additives [15,16]. To improve the recovery rate of Ti from VTMCs, the Ti-enriched slag was obtained by the direct reduction-magnetic separation process [17]. Because of the direct reduction process, the phase of the Ti-enriched slag constitutes a complex mixture of compounds comprised of anosovite ((Mg_0.6_Ti_2.4_)O_5_), metallic iron, ferriferous oxide, and a small amount of silicon dioxide, thus increasing the difficulty of subsequent utilization of the Ti slag [18,19,20,21]. Bian et al. [22] investigated the separation efficiency of vanadium, titanium, and iron from vanadium-bearing titanomagnetite carried out by pressurized pyrolysis of ammonium chloride-acid leaching-solvent extraction process. The observed leaching rates of iron, vanadium, and titanium were 92.5%, 95.1% and 1.2%, respectively. Zhong B et al. [21] showed that the content of TiO_2_ in the Ti-enriched slag prepared by two-stage acid leaching could reach 92.5% with further removal of residual carbon. The phase structure of ilmenite in vanadium titanite concentrate was altered by the pretreatment process of the above two processes. However, the preparation process of titanium slag from vanadium titanite concentrate by mechanical activation and hydrochloric acid leaching has not been reported. The mechanical activation process can lead to the increase of disordered structure in minerals, thus improving the reactivity between minerals and leaching agents. Therefore, it is often used to improve the leaching rate of elements in minerals [23,24,25].

In this paper, the mechanical activation-hydrochloric acid leaching method was used to treat vanadium titanite concentrate for the first time. The mechanical activation of VTMCs was investigated to effectively enhance the leaching rate of valuable elements. The hydrochloric acid leaching behaviors of unactivated and mechanically activated V-bearing titanomagnetite concentrate were also investigated. It is expected that the findings of this study will help establish an efficient route in producing Ti-enriched slag from VTMC without altering the mineral structure of the ilmenite phase.

## 2. Experimental

### 2.1. Materials

**Figure 1:**
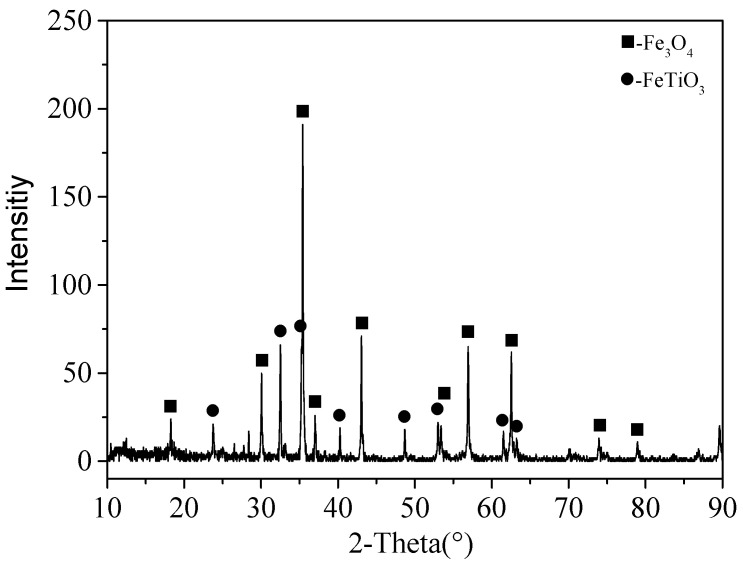
XRD of the VTMCs.

**Figure 2:**
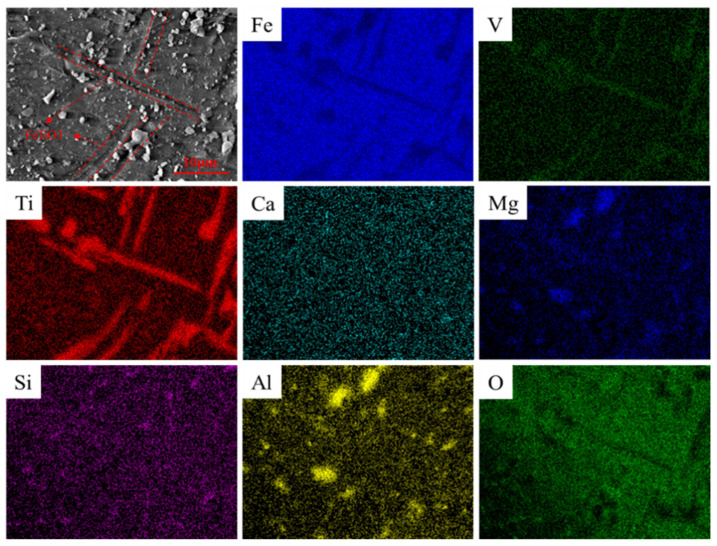
SEM images and element distribution maps of the VTMCs.

The VTMCs was obtained from the Sichuan Lomon Mining and Metallurgy Co., Ltd., Sichuan, China, and it was dried at 120 °C for 2 h. Its chemical composition, obtained with an X-ray fluorescence spectrometer, is presented in Table 1. The crystal structures and the element distribution maps of the VTMC were characterized using XRD and SEM, and the results are presented in Figure 1 and Figure 2, respectively. It is evident from Figure 1 that Fe_3_O_4_ (JCPDS card No. 89-2355) and FeTiO_3_ (JCPDS card No. 75-1208) are the main crystal structures in the VTMC. As shown in Figure 2, the width of the rod-shaped ilmenite is 1–3 μm, and the diameter of the granular magnesium–aluminate (Mg–Al) spinel is about 2 μm. The rod-shaped ilmenite and the granular Mg–Al spinel are distributed in the magnetite phase with a tabular and dense massive structure.

### 2.2. Experimental Procedures

The mechanical activation of VTMCs was performed in a vertical planetary ball mill (XQM-2, Changsha Tianchuang Powder Technology Co., Ltd., Changsha, China) comprising a steel cup and several steel balls. The milling conditions were: volume of the cup, 500 mL; milling atmosphere, air; milling rate, 200 rpm; ball to powder ratio, 10; activation time, 0–10 h. The milled samples were leached in a 1000-mL three-necked glass reactor. An agitator, a condenser, and a thermometer were fitted into its openings. The reactants were heated using a water bath which controlled the temperature within ±2 °C. In each experiment, 225 mL of 18% hydrochloric acid solution was preheated to the required temperature, and 25 g of the milled sample was added. During the entire leaching operation, the stirring speed, leaching temperature, and time were kept constant at 150 rpm, 90 °C, and 90 min, respectively. These optimization parameters of the acid leaching process refer to the literature [26].

### 2.3. Analytical Methods

The particle size distribution and specific surface areas of the VTMCs and milled products were determined using granulometric laser diffraction analysis (LPSA, Mastersizer2000, Malvern Instruments Ltd., Malvern, Worcestershire, UK). The micromorphology and element distribution maps of the VTMCs, milled products, and leaching residue were characterized via scanning electron microscopy (SEM, Quanta Q 400, FEI Company, Hillsboro, OR, USA). Their phase compositions were identified by an X-ray diffractometer (XRD, D8 ADVANCE, Bruker Company, Karlsruhe, Germany). The content of the total Fe and TiO_2_ in the leach residue was analyzed by potassium dichromate titration and ammonium ferric sulfate titration methods, respectively.

The sample was mixed with sodium borate and sodium carbonate as cosolvent and melted at a high temperature. The melt was decomposed by acid solution. The total iron content in the sample was titrated with potassium dichromate, combining with tin dichloride and titanium trichloride as reducing agents of ferric iron and sodium diphenylamine sulfonate solution as an indicator [27].

The sample and cosolvent were mixed evenly and melted at a high temperature using sodium borate and sodium carbonate as cosolvent. The melt was decomposed by acid solution, and the ferric iron was reduced by an aluminum sheet. Under the protection of sodium bicarbonate solution, ammonium thiocyanate solution was used as an indicator to titrate the content of titanium dioxide in the sample.

## 3. Results and Discussion

### 3.1. Structural Changes of the Mechanically Activated VTMCs

**Figure 3:**
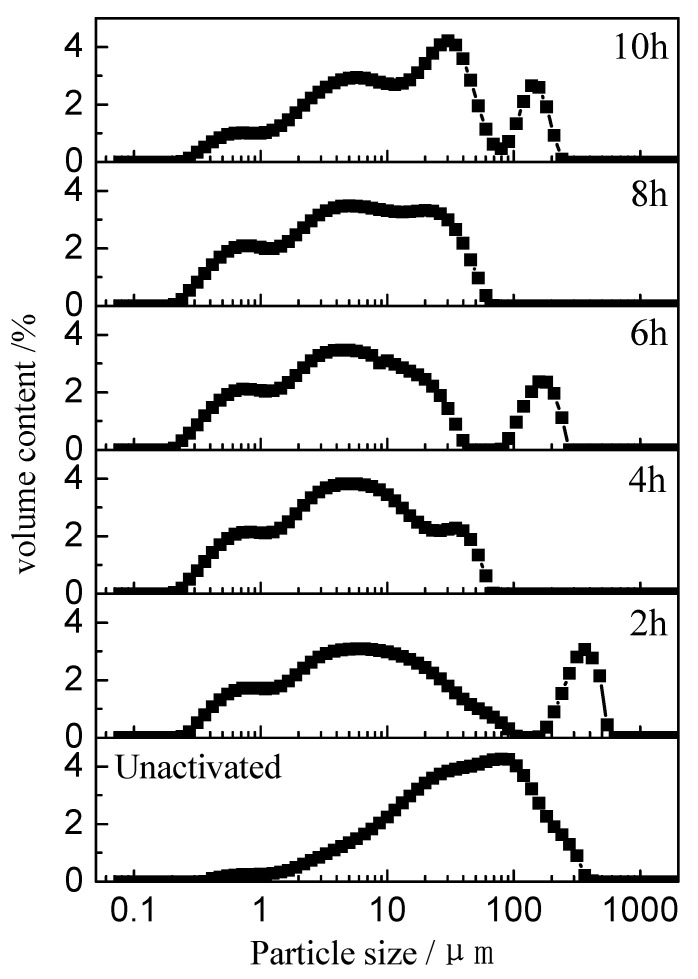
Particle size distributions of the activated and unactivated samples at different milling times.

**Figure 4:**
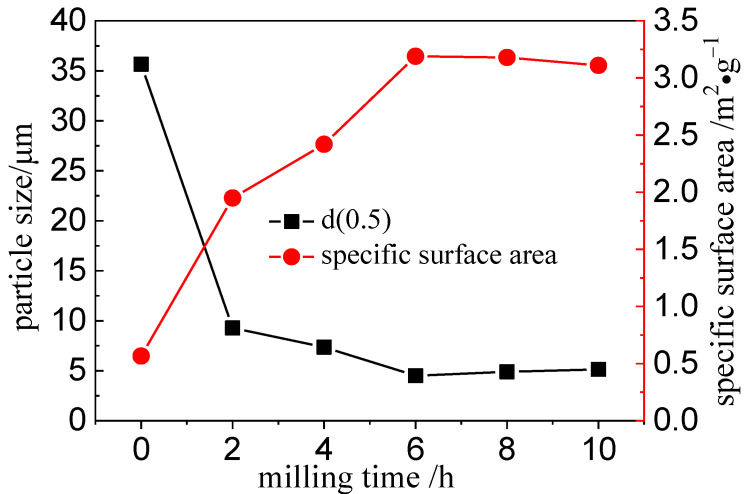
Changes in surface area and particle size with varying milling times.

The particle size distribution of the unactivated (0 h) and mechanically activated (2, 4, 6, 8, and 10 h) VTMCs samples are summarized in Figure 3. When the range of the milling time was 0 to 6 h, the particle size distribution shifted to the left as the milling time increased, and this indicated that an increase in the milling operation led to a gradual decrease in the particle size. However, when the milling time was prolonged beyond 6 h, the particle size distribution shifted to the right, and the particle size increased. The median volume particle diameter (d50) and the specific surface area (SA) are presented in Figure 4. From Figure 4, it is evident that d50 decreased, and SA increased rapidly when the milling time was below 6 h. However, when the milling time increased from 6 to 10 h, the d50 increased from 4.50 to 5.14 μm, and the SA decreased from 3.19 to 3.11 m^2^/g. The SA increased because an increase in the milling time (above 6 h) possibly led to agglomeration of the particles, which is consistent with the literature [28].

**Figure 5:**
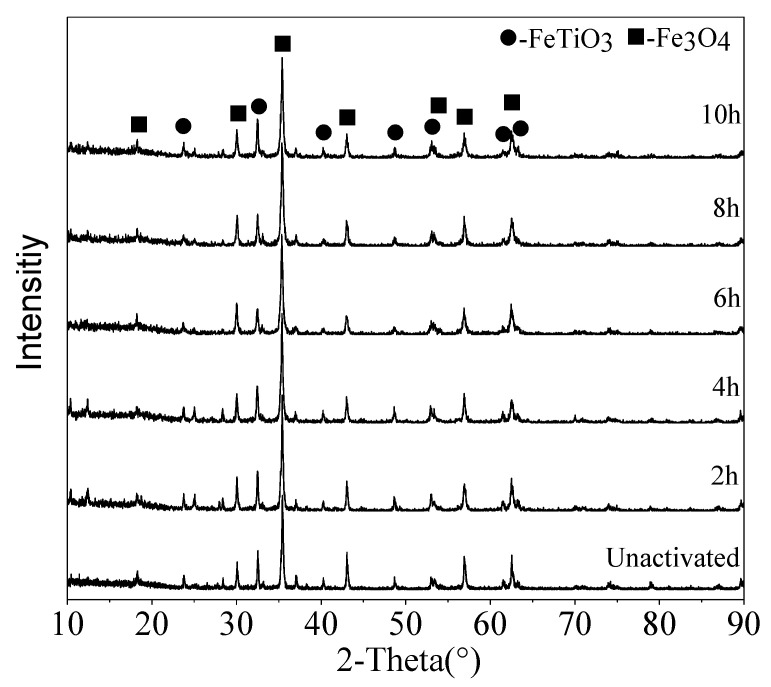
XRD patterns of the VTMCs mechanically activated at different times.

**Figure 6:**
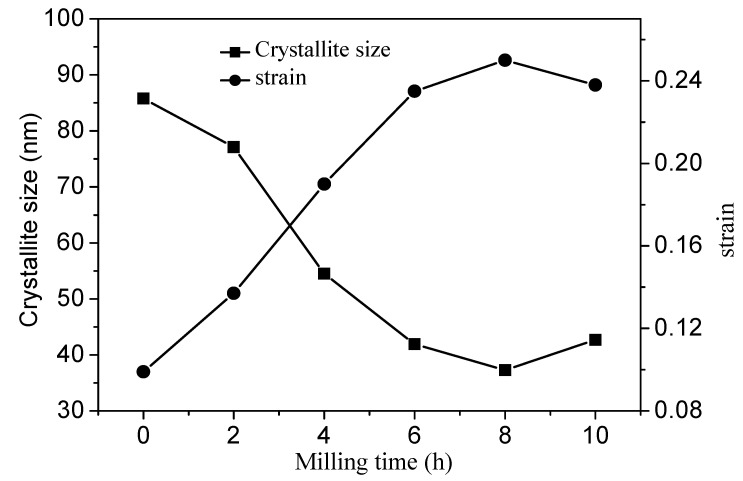
Variation in the crystallite size and strain with milling time.

The XRD patterns of the unactivated and activated VTMCs at different milling times are presented in Figure 5. As shown in Figure 5, the main diffraction peaks of the VTMCs are those of Fe_3_O_4_ and FeTiO_3_. There was no new phase formation in the samples during the ball milling process. It was observed that the intensities of the XRD diffraction peaks decreased slightly, and the line widths of the diffraction peaks widened marginally with an increase in the milling time in the range of 2–4 h. The recorded XRD spectra were used to evaluate the lattice strain and crystallite size from changes in the profile of the prominent peaks of the magnetite. The results of the evaluation are presented in Figure 6. It is evident that the crystallite size decreased, and the lattice strain increased with an increase in the milling time. 

**Figure 7:**
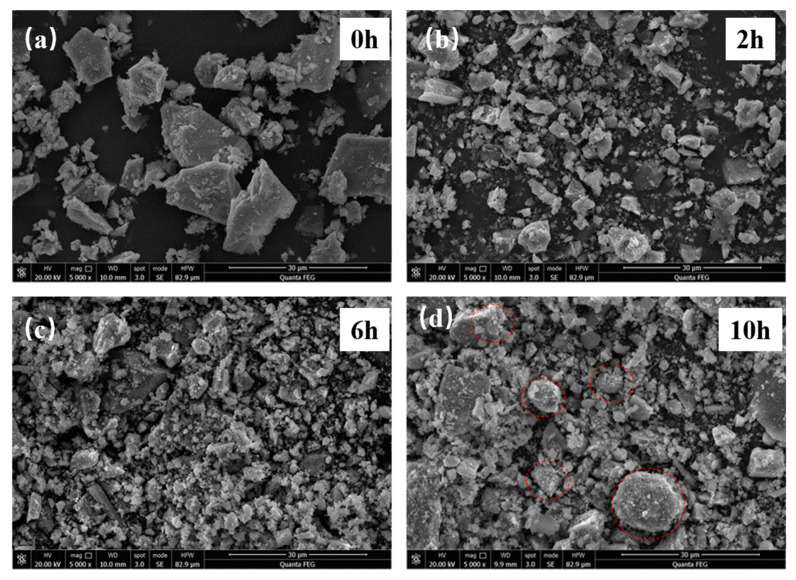
SEM images of the samples milled for different time (**a**) 0 h, (**b**) 2 h, (**c**) 6 h, (**d**) 10 h.

The morphological changes of the samples before and after the mechanical activation under different milling times are illustrated in Figure 7. The unactivated VTMCs comprised many particles formed by a large number of polyhedrons with varying sizes. The particles have been extremely ground, and their size decrease can be observed at 2 h. At longer milling times, the sizes of the particles further decreased. Upon reaching 10 h milling time, there was a slight agglomeration of the particles, and a couple of lumps with some surface agglomerations were evident. Figure 7 provides a visual explanation for the changes in SA and d50 when the milling time increased.

### 3.2. Hydrochloric Acid Leaching Behavior of Unactivated and Activated PTVMCs

**Figure 8:**
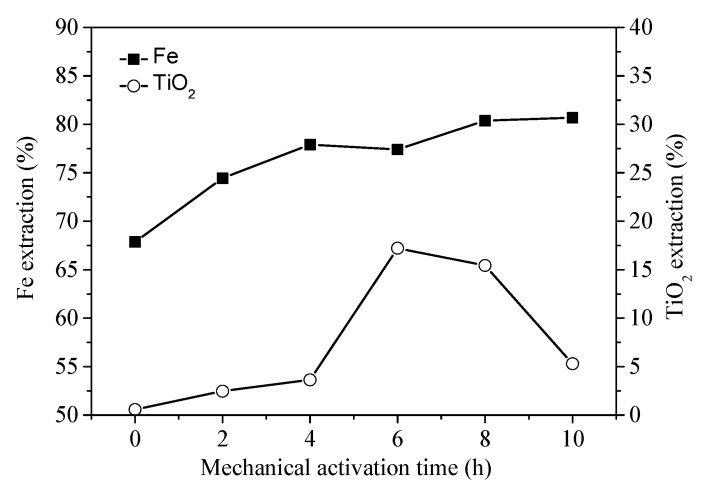
Rate of iron and titanium dioxide extractions with different mechanical activation times.

To investigate the influences of mechanical activation on the hydrochloric acid leaching behavior of Fe and Ti from the unactivated and mechanically activated VTMCs, all the leaching experiments were performed at 90 °C for 1.5 h. The results are shown in Figure 8, and they indicate that an increase in the mechanical activation time can enhance the solubility of Fe in the mechanically activated samples. However, the leaching rate of TiO_2_ initially increased and then decreased with added mechanical activation time. The possible reason for this phenomenon is that the TiOCl_2_ of the leaching solution can be hydrolyzed to produce the TiO_2_·nH_2_O and form precipitation in the leaching slag [29]. The hydrolysis reaction is presented in Equations (1)–(3).

Fe_3_O_4_ + 8HCl = 2FeCl_3_ + FeCl_2_ + 4H_2_O (1)

FeTiO_3_ + 4HCl = TiOCl_2_ + FeCl_2_ + H_2_O (2)

TiOCl_2_ + (*n* + 1)H_2_O = TiO_2_·nH_2_O↓ + 2HCl (3)

Considering the mechanically activated sample after 10 h, the Fe and TiO_2_ extraction from the activated sample compared to that of the unactivated sample was increased by 12.82% and 4.73%, respectively.

**Figure 9:**
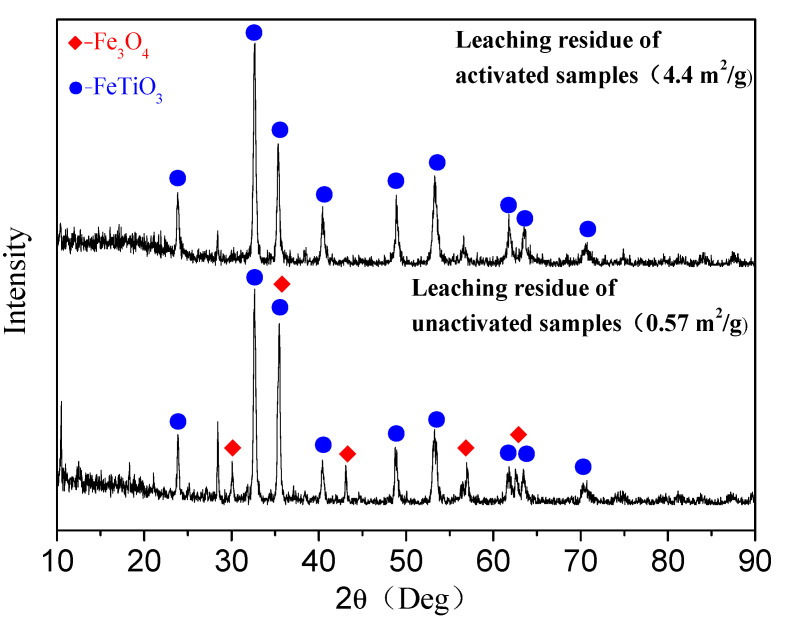
XRD patterns of the leaching residues for unactivated and mechanically activated samples.

The result of the XRD pattern of acid leaching residue is presented in Figure 9, where it can be deduced that the diffraction peak of the magnetite decreased after the acid leaching of the unactivated samples. However, the diffraction peak of the ilmenite increased, corresponding to that of the VBTC shown in Figure 1. The diffraction peak of the magnetite after the acid leaching of the mechanically activated samples at 10 h almost disappeared while that of the ilmenite further increased. It was remarkable that the phase of the ilmenite was not destroyed and remained in the leaching residue during the leaching process.

**Figure 10:**
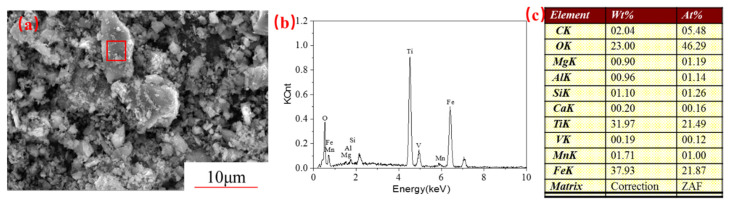
SEM image (**a**), EDS patterns (**b**) and area elements content (**c**) of the leaching residues sample.

Figure 10 shows the morphology of the leaching residues obtained when the concentrate was activated at 10 h. It is evident that the surface of the leaching residue of the mechanically activated sample was rough. The EDS shows that the peak height of the Fe decreased significantly, and that of Ti increased compared with the VTMCs. This observation indicates that the mechanical activation enhances Fe removal from VTMCs while the Ti is further enriched in the leaching residues. Additionally, the atomic ratio of Fe to Ti in the leaching residues is about 1:1, which further proved that the main phase in the leaching residues is ilmenite. The results of the chemical analysis of leaching residues are presented in Table 2. The TiO_2_ contents in the leached residue of the unactivated and activated samples were 32.19% and 43.75%, respectively. Comparing the enrichment of titanium slag in this work and the titanium concentrate in the Panxi area, the iron level contents and the TiO_2_ in the enrichment of titanium slag are similar. Acid oxide in the enrichment of titanium slag is higher than the titanium slag, and the alkaline oxide is even lower; thus, the enrichment of titanium slag can be used in the production of high titanium slag, and titanium white part of the raw material for using sulfuric acid method.

## 4. Conclusions

1. An effective technique to obtain Ti-enriched slag from mechanically activated VTMCs is atmospheric hydrochloric acid leaching.
2. Mechanical activation causes a decrease in the median volume particle diameter (d50) and an increase in the specific surface area (SA) with an increase in the milling time. The XRD patterns and SEM were conducted, and they confirmed the effects of the mechanical activation.
3. It was also demonstrated that mechanical activation could be employed to improve Fe recovery from VTMCs followed by hydrochloric acid leaching.
4. Moreover, the content of the TiO_2_ in the Ti-enriched slag can reach 43.75%, and the XRD patterns, as well as EDS, confirmed that the major phase in the Ti-enriched slag is the ilmenite.

## Figures and Tables

**Table 1:** Chemical composition of the vanadium–titanium magnetite used (wt.%).

TFe	TiO_2_	V_2_O_5_	Cr_2_O_3_	MgO	CaO	Al_2_O_3_	SiO_2_	MnO	SO_3_	K_2_O	NiO	ZnO
49.96	13.96	0.87	0.45	1.12	1.04	3.00	7.36	0.45	0.08	0.10	0.05	0.03

**Table 2:** Chemical composition of the leaching residues (wt. %).

Chemical Composition	TFe	TiO_2_	Cr_2_O_3_	MgO	CaO	Al_2_O_3_	SiO_2_	MnO
leaching residues with unactivated (0.57 m^2^/g)	35.5	32.19	0.25	0.54	1.33	1.57	11.92	1.08
leaching residues for activated (4.4 m^2^/g)	28.04	43.75	0.20	0.40	1.30	4.53	10.86	1.64
Commercial product	30.73	44.62	-	5.47	2.06	2.55	3.25	0.73

## Data Availability

The data presented in this study are available on request from the corresponding author.
